# Rheological evolution of a trachybasalt from Mt. Etna under slow cooling

**DOI:** 10.1038/s41597-026-07048-y

**Published:** 2026-03-13

**Authors:** Fabrizio Di Fiore, Alessandro Vona

**Affiliations:** 1 Istituto Nazionale di Geofisica e Vulcanologia, Sezione di Roma 1, Via di Vigna Murata 605, 00143 Roma, Italy; 2 Dipartimento di Scienze, Università degli Studi Roma Tre, L.go San Leonardo Murialdo 1, 00146 Roma, Italy

## Abstract

Understanding the rheological evolution of solidifying magma during its migration through the crust and the subsequent emplacement as lava is critical for assessing volcanic hazards. Crystallization plays a primary role in governing the rheology of low-viscosity basaltic magmas, thereby controlling lava inundation potential. While several studies have addressed this behavior under faster cooling conditions, experimental data bridging the gap toward the quasi-equilibrium regime remain scarce due to the technical challenges of long-duration high-temperature rheometry experiments. This highlights the critical need for new datasets designed to quantify the rheological evolution pertinent to lava flow emplacement conditions. Here, we present a rheological dataset carried out on an Etnean trachybasalt, focusing on low cooling rates (0.1 and 0.5 °C/min) under variable shear strain rates (1–10 s^−1^). Within the range of cooling and shear rates applied, results indicate that the cooling rate exerts a first-order control on crystallization kinetics, whereas the shear rate plays a secondary role, consistent with previous literature data. The technical validation is provided through instrument calibration and the verification of the chemical integrity of the pre- and post-run sample. Interestingly, the dataset captures the non-linear dependence of the crystallization onset temperature, which asymptotically approaches the thermodynamic liquidus (~1210 °C) as the cooling rate decreases. Beyond improving our understanding of magma crystallization kinetics, this dataset provides critical constraints for parameterizing the rheological evolution of lava flows during their emplacement in numerical models under varying thermal and dynamic regimes.

## Background & Summary

Viscosity is a fundamental physical property that controls magma transport, chemical differentiation, and eruption dynamics^1–9^. By regulating magma ascent rates, flow behavior within the crust and during lava emplacement, and the efficiency of gas segregation, viscosity ultimately dictates the style of volcanic eruptions^10–17^. Magmas are frequently subjected to rapid changes in temperature and pressure, which promote disequilibrium crystallization, while they simultaneously experience variable shear strain rates during flow. In such magmas, the interplay among nano- to microlite crystallization under disequilibrium conditions, residual melt compositional and structural evolution primarily controls the macroscopic rheological behavior^3,15,18–30^.

Several previous studies have shown that when mafic magmas are subjected to disequilibrium conditions that induce cooling and crystallization, the viscosity of the crystal-bearing suspension increases non-linearly, often by several orders of magnitude, fundamentally altering the capacity of the melt-crystal mixture to flow and its mechanical response to deformation^15,18,20,21,31,32^. This pronounced reduction in the ability of magma to dissipate stress through viscous flow promotes the onset of non-Newtonian behavior, which can lead to a shift in the eruptive style during magma migration through the Earth’s crust or affect the emplacement dynamics of a lava flow^6,13,16,33^.

Consequently, there is a critical need for experimental data that quantify the transient rheological evolution of magma, while systematically accounting for the complex interplay between cooling rates (*q*), shear rates ($\dot{\gamma }$), and the resulting crystallization kinetics under disequilibrium conditions.

To address this, rotational rheometry, most commonly performed using a concentric cylinder (CC) geometry, represents one of the most suitable experimental approaches. This technique allows real-time monitoring of changes in the apparent viscosity (*η*_*a*_) of the suspension in response to crystallization, which is tightly linked to the thermo-mechanical path experienced by the melt^3,21,28,34,35^.

To explore the solidification of magma under disequilibrium conditions, the experimental strategy commonly adopted is the Cooling Deformation Experiment(s) (CDE) during which the melt is cooled at a constant cooling rate (*q*) and shear rate ($\dot{\gamma }$) pertinent to magma migration and lava flow emplacement dynamics^7,8,18,21,31,36,37^. The interpretation of these rheological data allows us to better quantify the distinct roles of *q* and $\dot{\gamma }$ on the solidification paths of magmas. In addition, the parameterization and re-utilization of such data are essential for developing and calibrating more sophisticated numerical models that can accurately simulate magma ascent in conduit flow and lava emplacement scenarios^8,15,18,21^.

Here, we present a dataset of rheological measurements from CDEs performed on a trachybasaltic lava from the 2001 Mt. Etna eruption. This dataset provides 6 new rheological measurements detailing the evolution of apparent viscosity as a function of temperature adopting two distinct cooling rates (0.1 and 0.5 °C/min) and three different shear rates (1, 5, and 10 s^−1^). These data are intended to be reused by the community i) for understanding the concurrent role of cooling rate and shear rate on the solidification under disequilibrium kinetic conditions and ii) for parameterizing the rheological evolution of magma/lava flows and its timescales through more accurate numerical models.

## Methods

### Starting material, sample preparation, and chemical analysis

A trachybasaltic sample of lava from 2001 Mt. Etna eruption^38^ was utilized as starting material. Firstly, the rock sample was crushed and powdered using a jaw crusher and ring-mill. The resulting powder was then melted using a Fe-presaturated Pt crucible under air oxidizing conditions and ambient pressure in a Nabertherm® furnace at 1400 °C installed at the Experimental Volcanology and Petrology Laboratory (EVPLab) of the University of Roma Tre in Rome (Italy). In detail, the powdered sample was added to the crucible in batches of approximately 10–15 g every 10 minutes, to avoid the overflow of the melt due to foaming. When the crucible was completely filled, the melt was held to the target temperature of 1400 °C for approximately 2 hours to ensure the complete removal of bubbles and the melting of any pre-existing crystals. Subsequently, the melt was rapidly quenched in air to form a glass.

For the viscometry measurements, an aliquot of these glass chips was loaded and re-melted in a specific Fe-presaturated Pt_80_Rh_20_ cylindric crucible with the following dimensions: 62 mm height, 32 mm inner diameter and 1.5 mm wall thickness. The chemical analysis was carried out using a Jeol JXA-iSP-100 electron probe microanalyzer (EPMA) equipped with five wavelength dispersive spectrometers at the High Pressures–High Temperatures Laboratory of Experimental Volcanology and Geophysics (HP-HT Lab) of the Istituto Nazionale di Geofisica e Vulcanologia (INGV) in Rome (Italy). In detail, the analyses were carried out on carbon-coated samples under high vacuum conditions, using an accelerating voltage of 15 kV and an electron beam current of 10 nA, with a beam diameter of 5 μm. Elemental counting times were 10 s on the peak and 5 s on each of the two background positions. Corrections for interelemental effects were made using a ZAF (Z: atomic number; A: absorption; F: fluorescence) procedure. Calibration used a range of standards from Micro-Analysis Consultants (MAC; https://www.macstanda rds.co.uk): albite (Si-PET, Al-TAP, Na-TAP), forsterite (Mg-TAP), augite (Fe-LIF), apatite (Ca-PET), orthoclase (K-PET), rutile (Ti-PET), and rhodonite (Mn-LIF). To avoid alkali migration effects, Na and K were analyzed for first. BCR-2 was used as quality monitor standard during the analyses. Based on counting statistics, accuracy was better than 1–5%, except for elements with abundances below 1 wt%, for which accuracy was better than 5–10%. Precision was typically better than 1–5% for all analyzed elements. The chemical composition of the starting material is reported in Table 1.

**Table 1:** Chemical composition of starting material and post-run glasses.

Oxide	*Starting Material*		*Post CDE runs*	
	wt. %	s.d.	wt. %	s.d.
SiO_2_	47.39	0.24	47.51	0.28
TiO_2_	1.63	0.03	1.64	0.05
Al_2_O_3_	16.58	0.20	16.64	0.22
FeO	10.38	0.18	10.29	0.19
MnO	0.17	0.01	0.17	0.02
MgO	6.30	0.08	6.33	0.09
CaO	10.99	0.00	10.92	0.23
Na_2_O	3.32	0.21	3.25	0.07
K_2_O	1.90	0.06	1.84	0.05
P_2_O_5_	0.46	0.02	0.48	0.03
***VFT paramenters***		***A***	***B***	***C***
		−5.1	6321	553.2

Data are reported in wt.% and correspond to the average of 10 analyses. The VFT fitting parameters are also reported from **Di Fiore** ***et al****.*^7^.

### Cooling deformation experiments (CDE)

Cooling Deformation Experiments (CDE) were carried out using a Concentric Cylinder (CC) apparatus at EVPLab. The CC apparatus includes a Rheotronic II Rotational Viscometer (Theta Instruments) with an Anton Paar Rheolab Qc viscometer head (full-scale torque of 75 mNm), a Fe-presaturated Pt_80_Rh_20_ cylindric crucible (62, 32, and 1.5 mm in height, inner diameter, and wall thickness, respectively) and a Fe-presaturated Pt_80_Rh_20_ spindle (3.2 and 42 mm in diameter and length, respectively). The temperature of the sample was constantly monitored with a factory-calibrated S-type thermocouple (with a precision of ±2 °C) placed in close proximity to the crucible wall at the mid-height. In addition, the location of the thermocouple close to the sample permitted us to constantly monitor the absence of thermal lag in the sample during the experiments. The calibration of the CC apparatus was performed by using the standard glass NIST 717a (see *technical validation* section below).

**Fig. 1:**
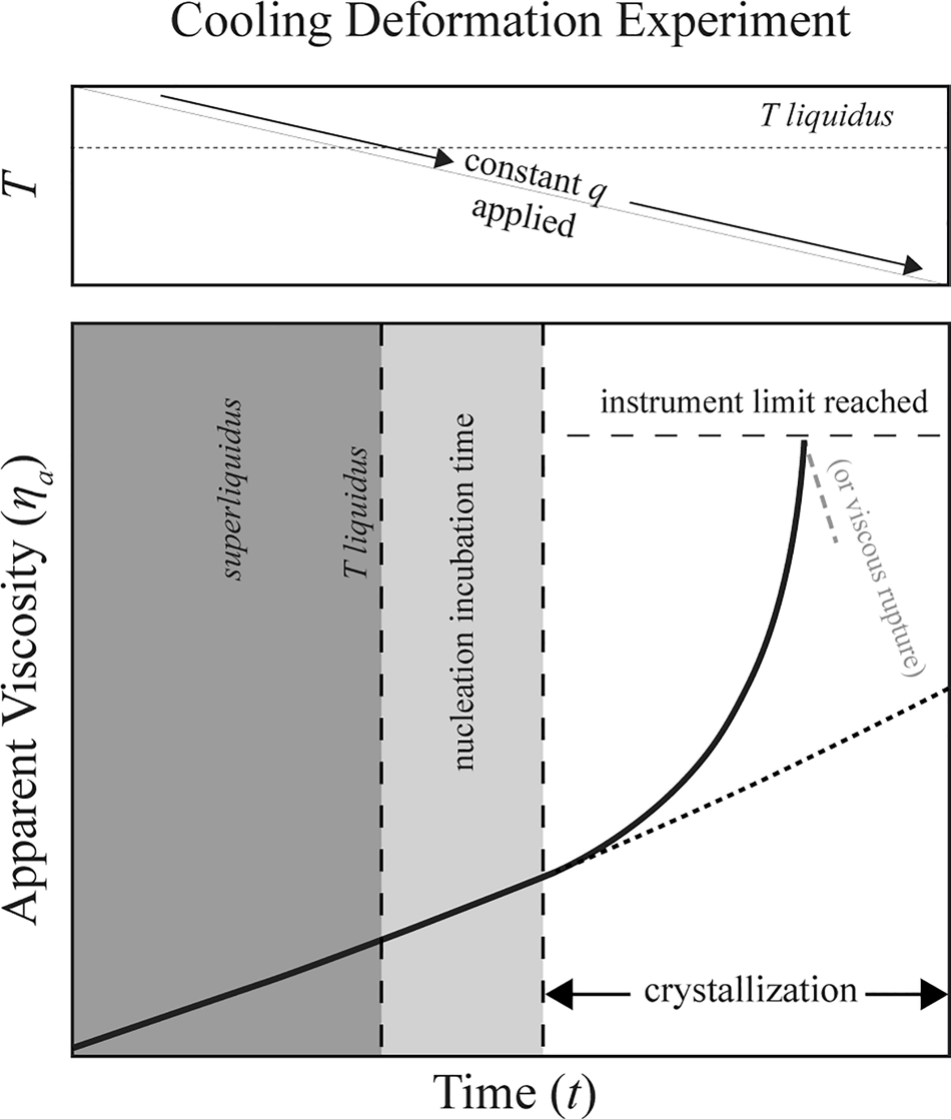
Schematic representation of the rheological path during a Cooling Deformation Experiment (CDE) (modified from^6^). The grey shaded area represents the *superliquidus* field. Upon cooling below the *liquidus* temperature, the system enters in the nucleation incubation zone. The onset of crystallization corresponds to the deviation of the measured apparent viscosity (solid line) from the theoretical pure liquid trend (dashed line). The experiment continues until the instrument torque limit is reached or viscous rupture occurs.

For the CDE presented in this study the cooling rate (*q*) adopted are 0.1 and 0.5 °C/min. For both *q*, a shear rate ($\dot{\gamma }$) of 1-5–10 s^−1^ are adopted. The range of *q* values reflects the thermal evolution typical of basaltic lava flows during emplacement and/or shallow transport, where cooling is primarily governed by conductive heat loss^8,21^. The adopted $\dot{\gamma }$ values are likewise representative of mafic lava transport and emplacement, particularly in high-strain near-boundary and localized shear zones within advancing flows, where $\dot{\gamma }$ can locally approach the upper limit of our experimental range^8,15^. The CDE protocol begins with a *superliquidus* treatment aimed at fully homogenizing the melt. The sample is heated to a temperature of 1400 °C at a rate of 30 °C/min^7^. When the temperature is reached, the melt is stirred at $\dot{\gamma }$ of 10 s^−1^ approximately 3 hours, until a constant viscosity is recovered (within ± 3% of the value related to the temperature). After this step, the experiment begins by applying to the melt a fixed *q* and $\dot{\gamma }$. The sampling frequency during each CDE is 60 seconds (s). The apparent viscosity (*η*_*a*_) of the melt follows the viscosity-temperature path of the crystal-free melt at *superliquidus* temperatures (Fig. 1). In this study, the crystal-free melt viscosity (*η*_*liquid*_) is modeled using the VFT equation^39^ as: Log *η*_*liquid*_ = -A + [B/(*T*_*k*_-C)], where *T* is the temperature in Kelvin. In detail, for this trachybasaltic melt the crystal-free melt viscosity was already measured in a previous work^7^ and the VFT fit-parameters are reported in Table 1. Once the temperature decreases below the *liquidus*, the melt continues to follow the pure liquid over the incubation phase, until the crystallinity of the suspension is sufficient to influence the rheology of the system (Fig. 1).

To identify the temperature of crystallization onset (*T*_*onset*_) during CDE, we adopt the point at which the measured viscosity deviates from the *η*_*liquid*_ of 10^0.05^ Pa s followed by a monotonic positive upward trend in viscosity. This viscosity increase reflects the development of a crystal fraction of ~1.8–3.3%, as estimated from empirical suspension models that incorporate shear rate and mean crystal aspect ratio^3,40^, for aspect ratios of 1–6 and $\dot{\gamma }$ of 0.1–10 s−¹. At this point, the viscosity diverges from the theoretical pure liquid trend and continues to increase until the achievement of the maximum stress of the device (i.e., 10^5^ Pa) during the experiment or manually halted when a large viscosity drop is observed during measurement (Fig. 1). This behavior, termed viscous rupture, corresponds to ductile failure associated with pronounced shear localization within the crystal-bearing suspension^6,21,28^. After the interruption of the experiment, the experimental charge was reheated and the sample was again subjected to a *superliquidus* treatment before initiating another experimental run. The primary limitation of the CDE technique is related to post-experiment sampling. The combination of rapid crystallization kinetics and the high viscosities achieved makes it unfeasible to recover samples representative of the solidification history to which the melt was subjected, at best representing a snapshot of the final state^8,20,21^. This, in turn, hinders the chemical analysis of the residual melt or the textural characterization of the crystal population. At the end of the final experimental run, the crucible was emptied by remelting the sample at 1400 °C followed by rapid quenching. In addition, we evaluated the rheological impact of crystallization on the trachybasaltic melt from Mt. Etna using a normalized viscosity (*η*_*x*_,^7^). This value is derived by dividing the apparent viscosity (*η*_*a*_) measured during CDE by the viscosity of the crystal-free liquid (i.e., *η*_*x*_ = *η*_*a*_ **/** *η*_*liquid*_). It is important to clarify that the normalized viscosity is distinct from the relative viscosity (*η*_*r*_), which is calculated as the ratio of apparent viscosity to residual melt viscosity. In fact, the calculation of *η*_*r*_ is unfeasible for CDE measurements as it requires data on the evolving composition of the residual melt to estimate the residual melt viscosity. Therefore, *η*_*x*_ is used as a comprehensive metric that reflects the combined influence of both physical factors (i.e., the presence of crystals) and chemical factors (i.e., the compositional evolution of the residual melt) on the suspension’s rheology^16,21,37^.

## Data Record

The data are available on Figshare at the following 10.6084/m9.figshare.30910934.

The dataset^41^ consists of a single Microsoft Excel file named “raw_data.xlsx”, which contains the complete time-series data from the Cooling Deformation Experiments (CDE). The file is organized into two main worksheets:

1. **Experimental Summary (“Summary”):** this worksheet provides a summary table of the experimental runs, listing the specific Cooling Rate and Shear Rate applied associated with the dataset.
2. **Experimental Data (“Raw_Data”):** this worksheet contains the raw rheological measurements. The data are organized in a side-by-side columnar format, where each experimental run is presented in a distinct block of columns grouped by the applied cooling rate (i.e., 0.1 or 0.5 °C/min) and shear rate (i.e., 1,5 and 10 s^−1^).

### Variable definitions

Within the “Raw_Data” worksheet, each CDE contains the following four variables:

- Temperature (°C): the temperature of the sample measured over time during the cooling ramp.
- Time (s): the duration of the experiment, with *t* = 0 corresponding to the start of the data acquisition at *superliquidus* conditions.
- Log Viscosity (Pa s): the base-10 logarithm of the measured apparent viscosity (*η*_*a*_) of the suspension.
- Log Normalized Viscosity: the base-10 logarithm of the normalized viscosity (*η*_*x*_), calculated as described in “*2.2 Cooling Deformation Experiments (CDE)*” section.

## Technical Validation

### Instrument calibrations

The Concentric Cylinder (CC) apparatus was technically validated to ensure the accuracy of both viscosity and temperature measurements. Prior to calibrating the rheometer, we characterized the furnace to identify a stable hot zone, ensuring the experiments were free from appreciable thermal gradients (see^7,16^ for additional information). The CC apparatus was calibrated using the NIST 717a standard reference material, a borosilicate glass with Newtonian properties (*n* = 1.0). Calibration measurements were performed across a temperature interval of 896 to 1504 °C, covering a torque range of ~0.1 to ~70.0 mN m and resulting a in a viscosity range between ~10^1.1^ to ~10^4.6^ Pa s, using shear rates ranging from 1 to 10 s^−1^ (see^7,16^ for additional information). As previously mentioned, the placement of the S-type thermocouple in close proximity to the sample allowed for constant monitoring to verify the absence of thermal lag throughout the sample during CDE experiments. In detail, no thermal lag was observed within the system, ensuring reliable temperature control across the entire experimental range up to a *q* of 10 °C/min.

### Sample chemical integrity

A primary element in validating the CDE measurements was verifying that no chemical modification occurred in the trachybasaltic sample. EPMA analyses conducted before and after the experiments confirmed the chemical stability of the material. Firstly, we ensured that the chemical composition of the starting glass (Table 1) was consistent with previously published analyses for the 2001 Mt. Etna lava^7^, confirming that the prepared material was representative of the target composition. In addition, at the conclusion of the final CDE run, the experimental charge was remelted at 1400 °C, subjected to a final *superliquidus* treatment cycle, and then rapidly quenched. EPMA analyses performed on the resulting glass chips showed no significant alkali loss (Na and K) despite the high-temperature and long-duration experimental steps (Table 1).

### Results and data consistency

**Fig. 2:**
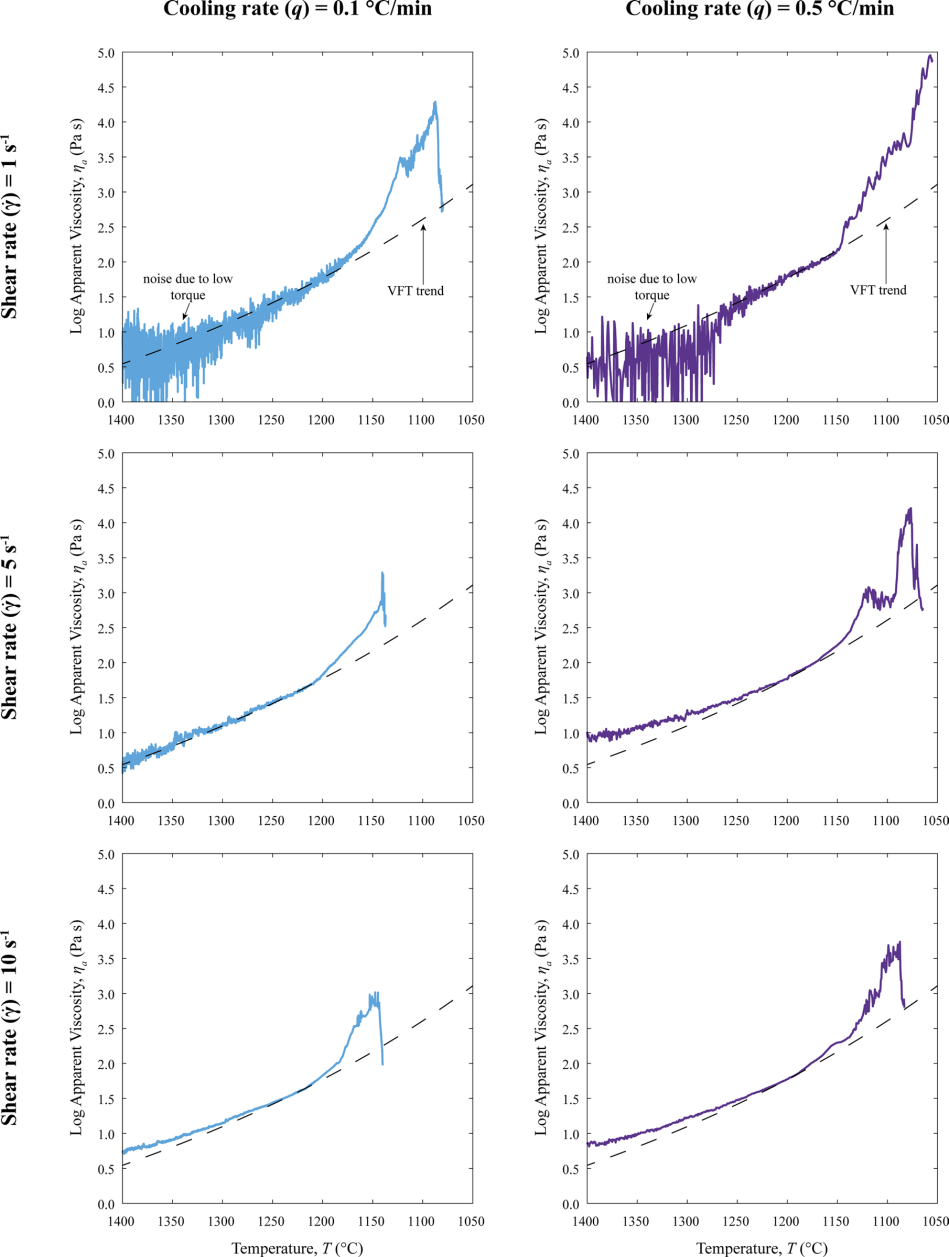
Evolution of apparent viscosity (*η*_*a*_) as a function of temperature (*T*). The plots show the CDE results for the trachybasaltic melt cooled at rates (*q*) of 0.1 and 0.5 °C/min, under shear rates ($\dot{\gamma }$) of 1, 5, and 10 s^−1^. The solid line represents the measured data, while the thin black line indicates the theoretical viscosity of the crystal-free melt (*η*_*liquid*_) modeled using the VFT equation (parameters in Table 1).

**Fig. 3:**
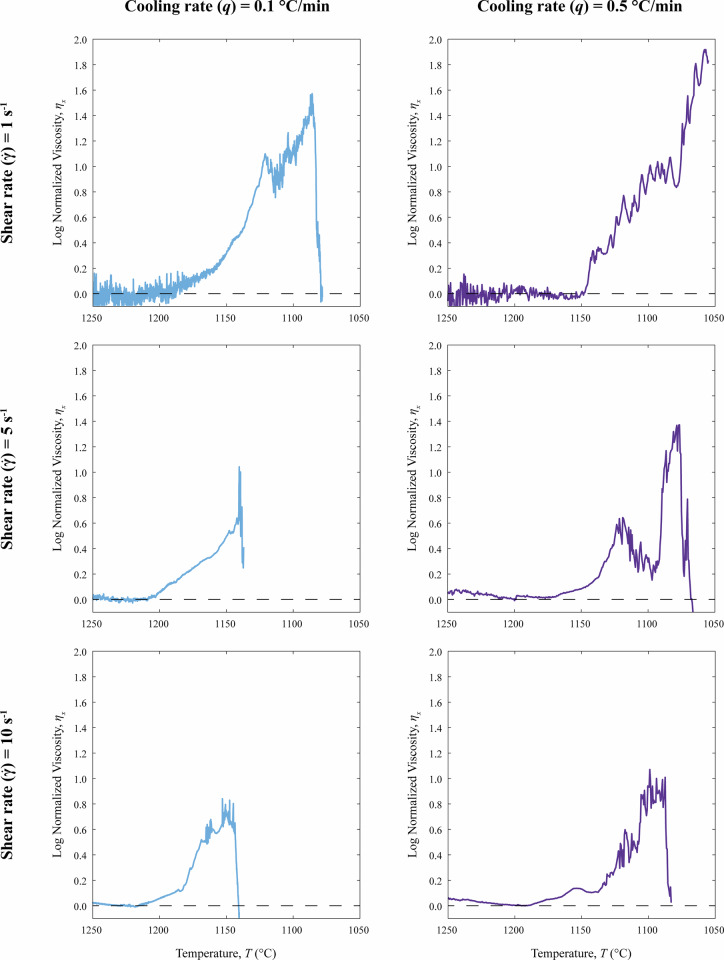
Evolution of normalized viscosity (*η*_*x*_) as a function of temperature (*T*). The *η*_*x*_ is calculated as the ratio between the measured apparent viscosity (*η*_*a*_) and the calculated pure liquid viscosity (*η*_*liquid*_). The curves highlight the rheological impact of crystallization under different disequilibrium conditions. Higher shear rates ($\dot{\gamma }$) and lower cooling rates (*q*) shift the viscosity increase to higher temperatures.

The viscosity data related to the CDE measurements carried out at different cooling rate (*q*) and shear rate ($\dot{\gamma }$) are reported as a function of temperature (*T*) in Figs. 2, 3. At *superliquidus T*, the apparent viscosity (*η*_*a*_) increases with decreasing *T* in all experiments. For CDE carried out at $\dot{\gamma }$ of 1 s^−1^ a noisy *η*_*a*_ signal is observed between 1400 and 1300 °C. This effect is attributable to the low torque recorded by the measuring system, resulting from the coupled effect of low $\dot{\gamma }$ and low *η*_*a*_ of the crystal-free melt at high *T*. Below 1300–1250 °C, once sufficient torque is reached for each $\dot{\gamma }$, in all CDE the viscosity increase trends are consistent with the evolution of the crystal-free melt viscosity modeled with the VFT equation, using the parameter reported in Table 1.

The temperature at which the crystallization takes place (*T*_*onset*_) was not chosen arbitrarily but was operationally defined as the point at which the measured *η*_*a*_ shows a deviation of 10^0.05^ Pa s from the modeled crystal-free melt viscosity (see Methods Section 2.2). All *T*_*onset*_ retrieved following this approach (see Table 2) are lower than 1210 °C that represents the *liquidus* temperature estimation of the starting chemical composition determined through the MELTS code^42^ in **Di Fiore** ***et al****.*^7^. The observed *T*_*onset*_ show higher values with decreasing *q* and increasing $\dot{\gamma }$. Notably, the experiment conducted at *q* = 0.5 °C/min and $\dot{\gamma }$ of 5 s^−1^ displays a non-monotonic rheological evolution (Fig. 2), characterized by a transient decrease in apparent viscosity (*η*_*a*_; up to ~0.2 log units). During this interval, the viscosity signal becomes noticeably noisier than during the rest of the experiment, within a temperature range of 1125–1100 °C. After this phase, viscosity increases rapidly, culminating in a large viscosity drop indicative of viscous rupture. Similarly, the experiment performed at *q* = 0.5 °C/min and $\dot{\gamma }$ of 10 s^−1^ exhibits a temperature interval between 1130 and 1110 °C characterized by a marked increase in viscosity signal noise, followed by a sharp viscosity increase. The occurrence of these comparable noisy intervals suggests that the observed rheological instability is likely related to shear-induced self-organization^43–45^, where high-aspect-ratio crystalline phases such as plagioclase tend to align with the flow direction^3,46–48^. Finally, consistent with the trend observed for *T*_*onset*_, the interruption of the experiments caused by the onset of viscous rupture^6,15,21^ also occurs at progressively lower *T* (from 1140 to 1077 °C Table 2) as *q* increases and $\dot{\gamma }$ decreases. The only exception is the experiment conducted at *q* of 0.5 °C/min and $\dot{\gamma }$ of 1 s^−1^, in which no viscous rupture was observed in the *η*_*a*_ interval accessible with our CC setup. In this case, the experiment was interrupted when the torque limit of the CC was achieved. The trends observed for both crystallization onset and viscous rupture are consistent with previously published rheological evolution patterns for similar mafic magmas subjected to comparable disequilibrium kinetic conditions in terms of *q* and $\dot{\gamma }$^6,7,15,16,20,21,31,36^.

**Table 2:** Results from Cooling Deformation Experiments (CDE).

*q*	$\dot{\gamma }$	*T*_*onset*_	*Tend*	*η*_*a*_	*η*_*x*_
°C/min	s^−1^	°C	°C	Log (Pa s)	Log units
0.1	1	1181	1087	4.30	1.56
0.5	1	1146	1055	4.86	1.91
0.1	5	1201	1140	3.29	1.01
0.5	5	1163	1077	4.20	1.37
0.1	10	1203	1143	2.99	0.81
0.5	10	1179	1088	3.73	1.01

Finally, the normalized viscosities (*η*_*x*_) of the CDE, plotted as a function of *T* in Fig. 3 highlight the impact of crystallization during the solidification of the Etnean trachybasalt under different disequilibrium conditions. The *η*_*x*_ values show a clear dependence on the experimental parameters, particularly, the onset of viscous rupture occurs at higher *T* and lower *η*_*x*_ values with decreasing *q* and increasing $\dot{\gamma }$. This behavior is consistent with previous findings reported in the literature^6,15,21,37^.

### Modeling the crystallization kinetics

**Fig. 4:**
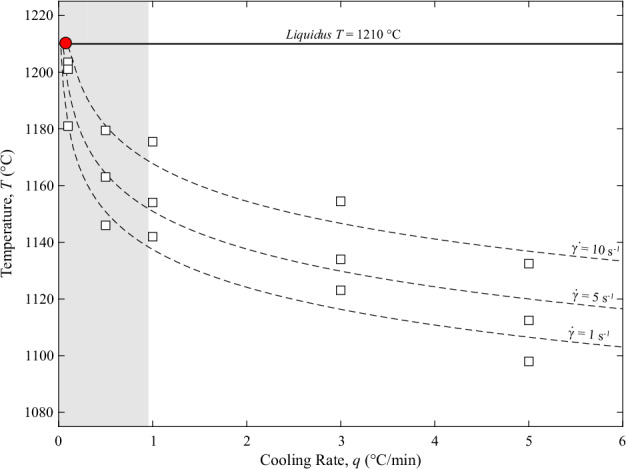
Crystallization onset modeling. (**a**) Crystallization onset temperatures (*T*_*onset*_) plotted as a function of cooling rate (*q*) for different shear rates ($\dot{\gamma }$ = 1, 5, 10 s^−1^). Symbols represent the experimental data from this study (low-*q* represented by the shaded grey field) integrated with high-*q* data from **Di Fiore** ***et al****.*^7^. Dashed lines represent the global regression fit based on the Lasocka relationship (*T*_*onset*_ = a – b × ln *q;*^49^). The red circle indicates the thermodynamic liquidus temperature (~1210 °C) estimated through the MELTS code^42^.

To assess the physical validity and consistency of the low-*q* experimental dataset presented in this study, we integrated our crystallization onset temperature (*T*_*onset*_) results with data previously reported by **Di Fiore** ***et al****.*^7^, in which higher *q* (from 1 to 5 °C/min) were investigated within the same $\dot{\gamma }$ range (from 1 to 10 s^−1^), adopting the same CC setup and geometries utilized in this study. The *T*_*onset*_ values corresponding to each *q* (and same $\dot{\gamma }$) were fitted using a global regression model based on the Lasocka relationship^49^ as follows:

1 $${T}_{onset}=a-b\times {\rm{l}}{\rm{n}}\,\,q$$

where *a* and *b* are fitting parameters (Fig. 4, respective values of *a* and *b* for each $\dot{\gamma }$ are listed in Table 2). The coefficients of determination (R^2^) for the fittings yield values ≥ of 0.94 (Table 3). Although Eq. 1 was originally developed to describe the rate-dependence of the glass transition temperature, this semi-empirical logarithmic relationship is widely used to model non-isothermal crystallization kinetics^50–52^, as both transformations are governed by time-dependent, thermally activated mechanisms.

**Table 3:** Summary of the fitting parameters (a, b) and determination coefficients (*R*^*2*^) for the Lasocka relationship describing the crystallization onset temperature (*T*_*onset*_).

*q*	$\dot{\gamma }$	*Fit Parameters*		*R*^*2*^
°C/min	s^−1^	*a*	*b*	—
0.1	1	1137.1	−19.267	0.95
0.5	1			
0.1	5	1151.4	−21.546	0.98
0.5	5			
0.1	10	1167.6	−16.874	0.94
0.5	10			

The fit was carried out on the integrated dataset comprising *T*_*onset*_ values from this work and from **Di Fiore** ***et al****.*^7^.

The observed trends, and specifically the kinetic delay of crystallization with increasing *q* and, conversely, the increase of *T*_*onset*_ with increasing $\dot{\gamma }$, are in excellent agreement with previous experimental studies on silicate melts carried out under similar experimental conditions^6,7,15,16,20,21,31,36^. In addition, extending the investigated range of *q* to values < 1 °C/min in this study, allows us to observe and quantify the non-linear dependence of *T*_*onset*_ for this silicate melt composition. In detail, with decreasing *q*, *T*_*onset*_ progressively approaches the *liquidus* temperature of 1210 °C, previously estimated in **Di Fiore** ***et al****.*^7^ on a thermodynamic basis through the MELTS code^42^. It is important to consider that while the rheological determination of the crystallization onset is subject to inherent experimental uncertainties related to the definition of the viscosity departure point, the coherence of the integrated fit across different dynamic conditions confirms the reliability of CDE measurements presented in this study and of the crystallization onset detection method.

## Data Availability

The data are available on Figshare at the following 10.6084/m9.figshare.30910934.

## Code availability

No custom code was used to generate or process the data described in the manuscript.
